# Headache from Eye Strain

**Published:** 1889-03

**Authors:** Wheelock Rider

**Affiliations:** Rochester, N. Y.


### AMERICAN JOURNAL

—OF—

### DENTAL SCIENCE.

VOL. XXII. THIRD SERIES—MARCH, 1889. NO. 11.

ARTICLE I.

### HEADACHE FROM EYE-STRAIN.

BY DR. WHEELOCK RIDER, ROCHESTER, N. Y.

[Read before the Medical Society of the County of Steuben, January 8th, 1889.]

That the symptom headache is of the greatest frequency few will deny. A large proportion of those seeking relief from the physician complain wholly or incidentally of headache, and devoid of danger as this disorder may be, its seriousness is by no means to be underated; in the aggregate probably more hours of suffering are annually caused by headache than by any other painful affection; not infrequently it necessitates a retirement from occupation or involves the loss of much time. Therefore, I trust a brief resume of the causal relations of refractive errors to headache, followed by a few illustrative cases, will be of interest to you.

Headache is best considered as arising by the combined existence of a general predisposing condition and a local irritation. Whatever this general condition may be, how brought about, or how continued, it is essentially a deficient resistence on the part of the general nervous system against the exciting causes. The periodically returning headache becomes, therefore, a veritable nerve storm set up by the occasional inability to resist an irritation, which in many instances, is continuously existing.

Both the predisposing condition and the cause of peripheral irritation are often inherited, a clinical fact of real value. Consumption, neuralgia, paralysis, etc., prevail in the family histories of many of these patients, as does strabismus, asthenopia and other evidences of defective vision. Among cases illustrative of this hereditary tendency let me mention one in which the attacks were very frequent, regularly recurring, severe headaches, accompanied generally by nausea, and the individuals affected were: mother, three sons, one daughter, two grandsons, and two granddaughters; in all the attacks were of a similar nature.

A word regarding supposed causation. There is rarely such uniformity among the laity in medical matters as is found in questioning these sufferers as to the supposed origin of their attacks. If it is not the liver to which the trouble is attributed, it is the stomach. Aside from the clinical evidence to the contrary it is extremely improbable that either of these organs can so universally be at fault. The idea arises in all probability from the observation that gastric function is imperfectly performed, and that vomiting frequently occurs during attacks. Much as in sea-sickness, the symptom so prominent is an effect, yet popularly the suffering member is credited with the offence. On the other hand the liver is a good scape-goat, its condition can not be easily demonstrated; appearance of bile in the stomach contents, resulting from prolonged vomiting, might also give rise to the belief that a disordered liver is the exciting cause.

Considering briefly the symptoms of sick headaches, it will be noticed that the special senses are abnormally acute; slight, and especially unpleasant odors are recognized with unusual promptness, hearing acuity is intensified, or rather, there is apparent hyperæsthesia rendering loud sounds very painful sometimes, and photophobia is very commonly present. Along with hearing may be mentioned the naturally associated vertigo, and subjective noises. Position most generally assumed therefore in severe attacks, is the horizontal one, in a darkened quiet room.

These few points are taken as bearing especially upon the particular cause I am to consider. Their incompleteness is, therefore, excusable. Let me recall briefly to your mind some matters in the physiology of vision.

During waking hours the eyes are at no time at rest; their eighteen muscles demand innervation sixteen hours daily. The slightest inequality of tension of the muscles governing the motions of the eyeballs produces marked discomfort, if uncorrected, double vision and vertigo, for the image of every object viewed, must, to be a clear one, fall directly upon the yellow spot of the retina; when both eyes are used—upon corresponding points. The smallest variation from the proper tension of the ciliary muscle on the other hand is productive of an indistinct image upon the retina, to avoid which, no effort seems too great.

In the normal eye, only parallel rays of light, those coming from a distance of about twenty feet or more, are focused on the retina. All clearer vision of nearer objects is the result of a greater or less amount of accommodative effort—the contraction of the ciliary muscle. But in viewing such near objects the visual axes cease to be parallel—the axes of vision converge toward each other—and this convergence of the visual axes sustains a definite relation to the amount of accommodation required for the given distance. An object at one foot requires a certain amount of effort of the ciliary muscle and a corresponding amount of effort of the internal recti muscles. At two feet both are changed in degree, but the same relation still exists. Nowhere else in the body is such exact relation muscular effort, such delicate adjustment required, and as habitually practiced.

The organ of vision in its more highly developed form, is the only organ in nature where perfect functional activity depends upon exact form—this is by reason of its dependence upon the laws of the refraction of light. The leaves of a plant or the teeth of an animal may be longer or shorter than the average and still each may be functionally perfect. An eye that is longer than the focal length of its refractive media is near-sighted and unable to conform completely to the requirements of the vertebrate eye. When too short it is over-sighted; here it can only be effective by exercising an effort of the ciliary muscle for distant vision —where the accommodation should physiologically be relaxed, and by excessive strain of the ciliary muscle when it is used for near objects. When irregularly curved, or where the ciliary muscle is irregularly exercised, its vision can be perfect only in exceptional cases. This latter condition is astigmatism.

Knowing as we do, the tendency to variation of form in nature, is it to be wondered at that abnormal optical conditions should so frequently be found, especially as we are able to put the eye to such a searching examination, that the slightest errors become manifest? As a matter of fact less than eight per cent. of a large number of eyes examined, were found by Randall strictly normal, optically.

Bearing now in mind the relation between convergence of the visual axes and accommodation, we see that hypermetropia at once introduces a disturbance of these relations. In distant vision with parallel axes the hypermetropic eye must accommodate—but parallelism and accommodation do not go together physiologically. In vision of near objects, also, as is readily seen, accommodation out of proportion to convergence exists, and the reverse obtains in myopia.

In the condition of regular astigmatism, one meridian is optically correct while another is not, or, both are at fault in different degrees, and the effort which corrects the one cannot correct both. All meridians cannot at once comply with the inviolable laws of light, expecting by an irregular contraction of the ciliary muscle—a feat of endurance.

In simple over-sight, then, the individual must:

I. Overwork constantly the ciliary muscle.

II. Violate at all times, the relation of convergence and accommodation.

When the patient has eyes of unequal refractive power —if he attempted binocular vision, he must be constantly striving to do what is next to impossible; correct at once, with the same force, eyes requiring different degrees of correction, else he must combine in the visual center a clear and an indistinct image.

In the case of astigmatism the individual is in a constant perplexity. No one effort of the ciliary muscle corrects for all meridians, and so this annular muscle is either obliged to contract irregularly or be constantly fretted by the demands made upon it. The convergence—accommodation relation is here also disturbed as is readily seen. The practical truth of these statements is nicely illustrated by the common defect of convergent strabismus. This, in the majority of cases, is a result of oversight or of defective vision of one eye. In the former case, the effort of the convergence-accommodation relation becomes so arduous that binocular vision is surrendered, as a lesser evil. If vision of one eye be markedly defective the organ is put in the direction of least resistance, and out of the way.

The patient never having enjoyed vision which is normal in acuity or ease, is generally ignorant of his defect. This is absolutely true in the majority of cases, though at first glance improbable.

Assuming now that a relation exists between the generation of nerve force, and its consumption in the exercise of duty of the parts of the organism, it would follow that an abnormal organ demanding, as it would, increased expenditure of nerve force to insure its physiological activity, would introduce an element of disturbance in supply and demand—and that where the demand exceeded the supply, symptoms such as headaches or neuralgias, might be developed as reflex symptoms of peripheral irritation.

The eye seems to be especially apt to introduce this disturbance by reason of prolonged constant use made of it, its tendency to fall short of its anatomical requirements, and the extreme nicety of adjustment demanded of it. As a clinical fact, however, defective teeth are a frequent cause of reflex irritation, and this appears to be more generally recognized in every-day treatment. So good an authority as T. Lauder Brunton states.* "The chief local causes are decayed teeth and abnormation of the eye." He narrates the case of a professional man who went upon a continental tour seeking relief from a headache incapacitating him from work. On his return, percussion of his teeth revealed one slightly carious and treatment of this immediately gave relief. In several of my own cases a relapse has been found to have resulted from the irritation of carious teeth of themselves painless, but the cause, in strict truth, of toothache in the back of the head.

Case I. Emma B., 21 years. Headache during childhood and school years. Gave up school because of headaches. Discovered while a child that glasses improved vision, and wore her mother's spectacles to improve her sight evenings, but her severe headaches were unaffected. Heaches are now developed during the day; pain across forehead and through temples. Nausea always, vomiting sometimes. Has been occupied six years in various department of book-bindery. Sick-headaches several times a week, lately daily. Never suspected eyes to be the cause

* "On the Pathology and Treatment of Some Forms of Headache." St. Bartholomew's Hospital Reports, Vol. XIX, 1883.

of headache. No asthenopia during the day, only by artificial light.

Vision right eye, 16-30, vision left eye, 16-80. With the ciliary muscle paralysed by a two per cent. solution of homatropine and cocaine, vision right eye, 16-50, vision left eye, less than 16-200.

Vision right, is 16-16 when corrected by + 2.75 s. ○ + 1. c. 70°.

Vision left, is 16-16 when corrected by + 1.50 s ○ + 1.25 c. 95°.

These lenses were ordered for all close work; complete relief has followed for the six months which have since passed. On one occasion, when deprived of her glasses for three days, a return of headache, severe in character, was noted.

The patient, whose history I now give, seems to have suffered severely from as slight a defect as is usually met with.

CASE II. C. N. W., 21 years, has always had headaches; remembers the interruptions in going to school when five or six years of age by reason of their occurrence. Attacks begin with blurring of vision, shortly followed by intense pain through temples and in occiput, with nausea and frequently vomiting. It matters little to what use the eyes have been subjected, whether to distant or near vision. Latterly he has wakened with headache. Pain will invariably follow an hour's close application. In driving along a country road the train of symptoms, as like as not, will set in and he will be obliged to get out of the carriage before vomiting begins. The most effectual treatment which he has discovered is the recumbent posture in a darkened room, with mustard foot-baths, and ice-bags on the head; has been confined many days at a time in this manner. He has consulted numerous physicians, but with no relief and has given up the study of a profession as impossible.

Was examined March 30, 1888. Vision 16-24 normal, with + .75 c., right 30°, left 150°; these lenses were ordered for constant use.

Four weeks later I found he had worn glasses with the greatest relief; had read six hours without producing discomfort; distant vision was steadier and head felt uniformly better. In a letter received from him eight months after he was first examined, he states: "It has now been five months since I have had a headache and I have had but three light attacks since I have worn the glasses." I may state that no other treatment was undertaken.

This patient believing his eyes normal and his liver rebellious had instituted a habit of diet marked by its frugality, which he believed to have lessened his attacks. Armed with his glasses I presume he is now omnivorous.

CASE III. Mr. H., a lawyer, about 45 years old, and in good general condition, became subject to attacks of headache about ten years ago. Both frequency and intensity gradually increased. The headache was liable to set in at any time, especially after close use of the eyes or after a walk in the bright sunlight, and the duration of an attack was variable. Nausea and vomiting later became prominent features in the case. Recumbency in a dark room with ice-bags on the head served to give some relief. His business was seriously interfered with and medical treatment had been of very little benefit, excepting to mitigate the severity of pain during attacks.

His eyes were examined in May, 1887. He was found to have a slight degree of astigmatism in the right eye, but a marked degree in the left one; the following correcting glasses were ordered for constant use:

Right eye, + .50 c. 90°.

Left eye, + 2. c. 90°.

During the twenty months which have passed since he began the use of glasses, he has not had a headache. No other treatment has been employed. Premonitory symptoms have appeared when at various times he has forgotten to put on his glasses before dressing in the morning.

This case exhibits a feature seen in many others, viz., the attacks were readily brought about by exposure to bright sunlight and sunheat. I have met with patients who referred the onset of their trouble to "slight sunstroke," and who thought themselves suffering from repeated mild insolations.

CASE IV. This case occurred in the practice of my friend, Dr. C. A. Dewey, who in sending the patient for examination furnished me the following notes:

"Kate D., 23 years old, single. Mother had paralysis at the age of 36. Has never been thoroughly well; had series of about 30 abscesses before the 18th year, which have scarred the left side of her neck. Seven years ago had rheumatism in ankles and wrists. Menstruated at 21, regularly; some pain in back and in ovarian regions. Bowels regular, urine normal. As a child was kept from school by sick-headaches. *Has always had neuralgia in right eye and forehead. The greatest length of continued freedom from this pain has been but a few weeks.* Uterus low, retroflexed, bound down by adhesions."

I found on examination, April 24, 1888, vision nearly normal. Under homatropine mydriasis, the following glasses were found necessary to produce normal vision:

Right eye, + .50 sph. ⊂⊃ 1. c. 90°.

Left eye, + 1.25 sph.

Ordered for constant use:

Right eye, + 1. c. 90°.

Left eye, + .75 sph.

For close work a weak prism (2°) was afterwards ordered in combination with the above, base inward.

This case is characteristic in that the pain was persistently located over and above the astigmatic eye. In the prescription an allowance for accommodative tension was made as is frequently desirable by omitting .50 D. sph.

This patient is of an extremely nervous disposition; largely confined indoors, and a recent attack of neuralgia had lasted several weeks when she was examined.

In this case the constant effort to correct a congenital optical defect induced the constant neuralgia of the ophthalmic division of the trifacial. No medication was used, the only other treatment being, latterly, application of a pessary.

Patient now is unable to dispense with the correcting glasses without evidence of the return of the habitual pain. Her efforts to obtain relief had been constant, but fruitless; no examination of the eyes had been suggested by the many physicians whom she had consulted, nor had she ever suspected defect of vision.

CASE V. Fannie M., 18 years, student. Headaches since earliest recollection. Severe sick-headaches brought on by any slight nervous excitement or close work; the excitement of attending places of amusement, receptions or in entertaining company provoked an attack; always considered delicate. No physician had ever suspected eye-strain. Two years ago suffered an extension of trouble down into neck and shoulders with pain and stiffness of these parts. At one period lost a number of days weekly by reason of her trouble, discontinued school for two months for same cause. For two years has had headaches daily, rarely vomiting, but nausea not infrequently. Pressing pain in vertex and across forehead; a tender spot on vertex makes combing of the hair painful.

Vision, each eye, 16-16, under homatropine, vision sub-normal; + .75 c. 90°, each eye, perfects it. Has had no further trouble in about six weeks following correction of the slight hypermetropic astigmatism. Occasionally wakes with headache which disappears shortly after putting on glasses. Has had a few repetitions of the trouble, but only when the glasses were not worn. No other treatment.

An uncorrected error of refraction will produce localized tenderness in various parts of the surface of the head. The wearing of weak convex cylindrical glasses, unsuited to the case, often produced in a few hours extreme tenderness in the center of the forehead, in a patient that came under observation. I repeatedly produced it.

I think the following case is interesting in view of the slight optical error which has produced such life-long discomfort.

CASE VI. Mrs. P., 24 years, married, no children. Examined February 18, 1888. Has been subject to a slight constant headache since earliest remembrance, location is described in my notes as "all over the head, sometimes above forehead." Has always considered her eyes perfect, expecting, when as she thought, the headaches of unusual severity caused a blurring of vision. Vision both eyes 16-12, accepts on a preliminary examination a weak convex lens (+ .50) under homatropine, vision slightly sub-normal, raised to normal in each eye with + .50 c. 90°. This was ordered for constant use. They have been worn nearly eleven months; vision with them is no sharper than without, in both cases being perfect, but ease of vision with the glasses is marked. The relief from constant headache was immediate and permanent.

In such cases as this it might be suggested that relief followed correction, by chance only; that the conclusion is not reached by insufficient induction is shown by ordering discontinuance of the glasses—pain is found to return in the habitual manner. This was once the case in my office while this patient was awaiting examination of a sister with a similar complaint. I removed the glasses for another patient's use; when I returned them a short time after, she assured me that her head was aching.

CASE VII. F. A. P., 35 years, groceryman. Is sent for examination by family physician, with a history of intense pain in head associated with vertigo. Trouble has existed nine months. It begins usually, not always, after close use of eyes, with blurring of vision then intense pain and *such dizziness as to cause a staggering gait, simulating intoxication if patient walks in the street.* No nausea. Never noticed defective vision.

Under homatropine, vision right, 16-40; left, 16-80.

Vision normal with + 1.50 c. 90° each eye. With this correction for close work only, *absolute and immediate relief for 21 weeks.* An occasional headache, not at all severe, now appears, and the patient is at present under observation. Either further correction is needed, or dental or other irritation is present.

No thorough examination of the refraction can be made in persons of an age under 40 years, without previously paralysing the accommodation. If this be left undone the results may be in some cases correct, are in many entirely misleading—in all, doubtful. The involuntary contraction of the ciliary muscle being the only element of uncertainty, as soon as it is eliminated, the problem becomes a very simple one. Small convenient test-cases of lenses are sufficient to enable any physician to establish beyond doubt refractive errors in such cases as come under his care. Many instances occur where special experience may be needed, however, to properly correct them.

This whole subject is no novel one; but it may be that of late it is being more habitually recognized by the profession, and neurologists especially. Brunton states, "In treating any case of headache, the first thing to do is to see whether the teeth are sound and the eyes normal." Again, "It would be going too far to say that frontal headache is always due to an abnormal condition of the eyes, but I believe it is so much more frequently that one would at all suspect."

The satisfaction with which I consider a considerable series of cases, of which I fear I have here detailed too many examples, is only equaled by surprise that the true character of the trouble has been recognized in so few instances; not until almost all other means had been tried over and over again.

To formulate briefly the subject-matter of this paper:

I. It matters little what be the occupation, sex, age or apparent perfection of vision of a patient, the eyes may be the cause of the headache or neuralgia, and the burden of proof lies on the other side of the proposition.

II. If an optical condition requires such effort, with its resulting irritation, as to produce the reflex it must be completely relieved, bearing in mind that a mydriatic is necessary in examination, and then the cure will be brilliant.

III. The most common origin of reflex irritation from eyes is oversighted astigmatism. The recent statement of a noted French authority, in publishing a series of several hundred of these cases, goes, as far as I can learn, unchallenged: "Astigmatism should be regarded as an almost universal cause of migraine."

IV. Blurring of vision at any time during an attack should immediately lead to an examination of the eyes.

V. The predisposing condition may be overcome, but for time being only. The local irritation, if eye-strain, can be overcome; and this is permanent.

This may all be familiar ground to you. If so, you can pardon the enthusiasm which leads a prominent neurologist to say, "I have never seen a case of typical sick-headache where the eyes themselves, or the eye-muscles, were not at fault."—*Odontographic Journal.*

